# Neuronal Nitric Oxide Synthase in Vascular Physiology and Diseases

**DOI:** 10.3389/fphys.2016.00206

**Published:** 2016-06-02

**Authors:** Eduardo D. Costa, Bruno A. Rezende, Steyner F. Cortes, Virginia S. Lemos

**Affiliations:** ^1^Department of Physiology and Biophysics, Institute of Biological Sciences, Universidade Federal de Minas GeraisBelo Horizonte, Brazil; ^2^Department of Health Sciences, Post-graduate Institute, Medical Sciences CollegeBelo Horizonte, Brazil; ^3^Department of Pharmacology, Institute of Biological Sciences, Universidade Federal de Minas GeraisBelo Horizonte, Brazil

**Keywords:** neuronal nitric oxide synthase, nitric oxide, hydrogen peroxide, vascular function, hypertension, atherosclerosis

## Abstract

The family of nitric oxide synthases (NOS) has significant importance in various physiological mechanisms and is also involved in many pathological processes. Three NOS isoforms have been identified: neuronal NOS (nNOS or NOS 1), endothelial NOS (eNOS or NOS 3), and an inducible NOS (iNOS or NOS 2). Both nNOS and eNOS are constitutively expressed. Classically, eNOS is considered the main isoform involved in the control of the vascular function. However, more recent studies have shown that nNOS is present in the vascular endothelium and importantly contributes to the maintenance of the homeostasis of the cardiovascular system. In physiological conditions, besides nitric oxide (NO), nNOS also produces hydrogen peroxide (H_2_O_2_) and superoxide (${\text{O}}_{2}^{•-}$) considered as key mediators in non-neuronal cells signaling. This mini-review highlights recent scientific releases on the role of nNOS in vascular homeostasis and cardiovascular disorders such as hypertension and atherosclerosis.

## Introduction

Since the early 80s, nitric oxide (NO) is considered an essential endothelium-derived molecule, crucial to the maintenance of cardiovascular homeostasis (Furchgott and Zawadzki, 1980). Later on, it became evident that a decrease in the bioavailability of NO participated in several cardiovascular disorders such as atherosclerosis (Napoli et al., 2006) and hypertension (Hermann et al., 2006).

NO is biologically generated by a family of three nitric oxide synthase enzymes (NOS) isoforms: neuronal nitric oxide synthase (nNOS or NOS1), inducible nitric oxide synthase (iNOS or NOS2), and endothelial nitric oxide synthase (eNOS or NOS3). Although nNOS is abundantly expressed in neurons, and associated with the control of neuronal functions (Bredt et al., 1990; Bredt and Snyder, 1992) it is known that this isoform is also expressed in many non-neuronal cells such as in the endothelium and smooth muscle cells of several types of vessels in animals (Boulanger et al., 1998; Loesch et al., 1998; Schwarz et al., 1999) and human (Buchwalow et al., 2002). Recent studies show consistent evidence that this isoform exhibits relevant physiological role in the control of vascular homeostasis (Kurihara et al., 1998; Fleming, 2003; Hagioka et al., 2005; Seddon et al., 2008, 2009).

Besides NO, nNOS also produces H_2_O_2_ in physiological conditions that contributes to endothelium-dependent vascular relaxation (Capettini et al., 2008, 2010). Impairment in endothelial nNOS-derived H_2_O_2_ production has been implicated in the endothelial dysfunction in atherosclerosis (Rabelo et al., 2003; Capettini et al., 2011) and hypertension (Silva et al., 2016). Given the importance of nNOS in health and disease, this mini-review highlights recent scientific releases on the role of nNOS in vascular homeostasis and vascular mal functioning linked to hypertension and atherosclerosis.

## Gene expression and molecular structure of nNOS

nNOS gene is positioned on chromosome 12 (12q24.2) and distributed over a region greater than 200 kb in human genomic DNA (Hall et al., 1994). It consists of 4299 nucleotides encoding 1434 amino acids (Boissel et al., 1998). nNOS exists as a monomer/dimer mixture, being the dimer the active form. Each monomer consists of two domains: N-terminal (catalytic or oxygenase) and C-terminal (reductase). The N-terminal domain binds to the thiolate-linked heme group, tetrahydrobiopterin (BH_4_), a redox co-factor; L-arginine the substrate, and the zinc ion. The C-terminal domain has binding sites for flavin mononucleotide (FMN), flavin adenine dinucleotide (FAD), and nicotinamide adenine dinucleotide phosphate (NADPH; Masters et al., 1996; Sagami et al., 2001; Feng et al., 2014).

## nNOS regulation

### Intrinsic factors

#### Auto-inhibitory domain and C-terminal tail

A sequence of 40–50 amino acids inserted in the FMN domain is related to nNOS auto-inhibition by destabilizing calmodulin (CaM) binding to the enzyme and inhibiting intra- and inter-module electron transferring. This interaction occurs in low intracellular Ca^2+^ concentration ([Ca^2+^]_i_), taking part in the modulation of nNOS activity (Salerno et al., 1997; Daff et al., 1999; Garcin et al., 2004). Similarly, nNOS has a tail sequence of 21–42 amino acids at the C-terminal, related to the enzyme inhibition. Removal of this extension results in increased transference rates of electron flow in the reductase domain (Roman et al., 2000). Deletion of the auto-inhibitory domain and C-terminal tail results in CaM-independent electron transferring through the reductase domain, despite CaM is still required to promote electron transference from the FMN domain to the heme for NO production (Roman and Masters, 2006).

#### Dimer stability

The dimerization maintained by the N-terminal domain is crucial for the catalytic activity of nNOS. Otherwise, the transport of electrons and formation of nNOS products do not exist (Stuehr, 1997). Dimer formation has the participation of residues from the oxygenase domain that form a “hook” which reaches across to the oxygenase domain of the other subunit to coordinate dimer formation (Crane et al., 1998). Zinc binding has a contribution in dimer stabilization (Hemmens et al., 2000). The disulfide bonds formed by cysteine residues along the nNOS molecule and BH_4_ binding are also important to stabilize nNOS dimeric form (Hemmens et al., 1998; Kamada et al., 2005).

### Extrinsic factors

#### Phosphorylation

Phosphorylation of nNOS has been shown to be the critical stage in the activation/inactivation of this isoform. Several phosphatases and kinases including protein kinase A, CaM-kinases (CaM-KI and CaMKII), protein kinase C, and phosphatase 1 may regulate the activity of nNOS. For instance, CaM-KI and CaM-KII phosphorylate Ser^741^ and Ser^852^, respectively, resulting in reduced activity of the enzyme through inhibition of CaM binding (Song et al., 2004). Phosphorylation on Ser^1412^ (in rat) or Ser^1212^ (in human) residue is associated with increased activity of nNOS (Chen et al., 2000; Adak et al., 2001).

#### nNOS uncoupling

The deficiency of L-arginine or BH_4_ may produce nNOS uncoupling and the enzyme synthesize superoxide instead of NO. Recently, it has been reported impaired NO signaling due to nNOS uncoupling in brain arteries of obese rats and consequent oxidative stress and vasoconstriction (Katakam et al., 2012). Moreover, nNOS uncoupling is associated with penile arteries constriction with erectile dysfunction in a model of metabolic syndrome (Sanchez et al., 2012).

#### Protein-protein interactions

Protein-protein interaction is one of the key events in controlling the enzymatic activity of NOS. There are numerous proteins that may have physical interaction with nNOS in a variety of roles including activation, inhibition, and trafficking within the cell.

#### Ca^2+^/CaM complex

The increase in [Ca^2+^]_i_ and its subsequent binding to CaM is the main modulatory event of nNOS activation (Bredt and Snyder, 1990). The first step of nNOS activation consists of binding Ca^2+^ in CaM C-terminal domain. In sequence, the CaM C-terminal domain binds to nNOS. Then, in a similar way, Ca^2+^ binds to the CaM N-terminal domain, which also binds to nNOS and causes the activation of nNOS by the displacement of the auto-inhibitory domain of the enzyme. When the [Ca^2+^]_i_ decrease, CaM dissociates from nNOS, and it becomes inactive again (Weissman et al., 2002).

#### Caveolin/caveolae

Caveolins are scaffolding proteins situated at the caveolae, the flask-shaped non-clathrin-coated invaginations of the plasma membrane (Sowa, 2012). In skeletal muscle, nNOS directly interacts with caveolin-3, involving two distinct and physically separated caveolin scaffolding domains. This interaction inhibits nNOS activity (Venema et al., 1997). In a rat model of myocardial infarction, nNOS upregulation is associated with an increased binding with caveolin-3 (Bendall et al., 2004). Moreover, caveolin-1 interacts with the oxygenase and reductase nNOS domains inhibiting electron transfers (Sato et al., 2004).

#### Protein inhibitor of nNOS (PIN)

The NH2-terminus of nNOS has a binding site for the protein PIN (Jaffrey and Snyder, 1996). This endogenous protein inhibits nNOS by destabilizing the dimer isoform. Curiously, some studies have shown that PIN plays a physiological role in the control of insulin secretion (Lajoix et al., 2006). Moreover, neurogenic erectile dysfunction (NED) may be caused by impairment of nNOS regulation by PIN (Gonzalez-Cadavid and Rajfer, 2004).

#### PDZ domain

The nNOS PDZ domain has 80–120 amino acid residues located in the NH_2_-terminus. The PDZ domain participates in the formation of active nNOS dimers and interacts with other proteins in different regions of the cell (Roman et al., 2002). A study to assess potential ligands for PDZ domain of nNOS was conducted by screening 13 billion different peptides and had found that this motif binds to peptides ending with Asp-X-Val.

## Formation of nNOS products

**Figure 1:**
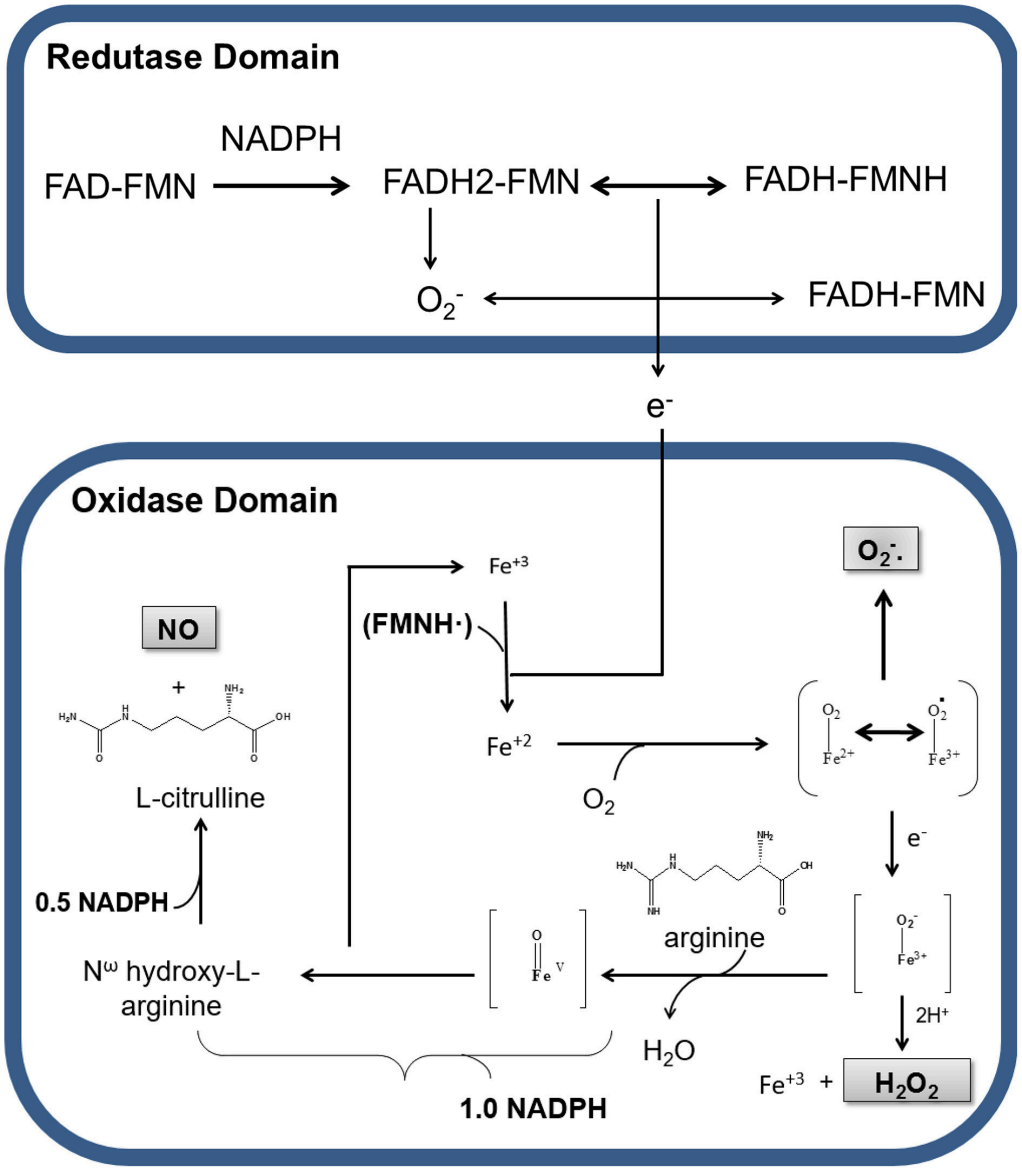
**Production of nitric oxide (NO), hydrogen peroxide (H_2_O_2_), and superoxide anion (${\text{O}}_{2}^{•-}$) by nNOS in physiological conditions**. Representation of electrons transport between the reductase and oxidase domains of nNOS. Nicotinamide adenine dinucleotide phosphate (NADPH) transfers electrons to the flavin adenine dinucleotide (FAD) and flavin adenine mononucleotide (FMN) in the reductase domain. In this process, oxygen receives electrons, being converted in superoxide (${\text{O}}_{2}^{•-}$). In the presence of Ca^2+^/CaM electrons from the reductase domain, enable nNOS Fe^3+^ to bind O_2_ and to form O_2_-Fe^2+^, in the oxidase domain. During electronic switching in the heme group, H_2_O_2_ is produced, with consequent release of Fe^3+^. In the presence of BH_4_ and NADPH, a nitrogen group is inserted into L-arginine, generating the intermediate N^ω^-hydroxy-L-arginine, which is there after transformed in NO and L-citrulline.

NO formation through L-arginine is catalyzed by nNOS in two steps: the hydroxylation of L-arginine to the intermediate N^ω^-hydroxy-L-arginine (NOHA), which is then oxidized to L-citrulline and NO (Papale et al., 2012). In the first step, NADPH transfers electrons to FAD and FMN, which have the capacity to reduce molecular oxygen to superoxide (${\text{O}}_{2}^{•-}$) (Figure 1). At the same time, an electron from flavin-mononucleotide (FMNH) reduces the heme group (Fe^3+^ to Fe^2+^). The reduction of Fe^3+^ enables O_2_ linking resulting in an ${\text{O}}_{2^{-}}$Fe^2+^ complex. The electron from the complex alternates between Fe^2+^ and O_2_, resulting in the complex ${\text{O}}_{2}^{•-}$Fe^3+^. In the deficiency of L-arginine or NOHA, ${\text{O}}_{2}^{•-}$Fe^3+^ transfers an electron to O_2_ liberating superoxide (${\text{O}}_{2}^{•-}$). Studies have revealed that the heme group of nNOS oxidase domain is responsible for 90% of ${\text{O}}_{2}^{•-}$ formation by this enzyme (Yoneyama et al., 2001). Alternatively, the intermediate ${\text{O}}_{2}^{•-}$Fe^3+^ can receive an electron, forming ${\text{O}}_{2^{-}}$Fe^3+^ that interacts with H^+^ and releases H_2_O_2_ and Fe^3+^.

In order to make the catalysis of L-arginine possible, BH_4_ cofactor must be binding to ${\text{O}}_{2}^{•-}$Fe^3+^ present in heme group. Electrons from BH_4_ cofactor are responsible for the formation of peroxy complexes (Fe^3+^-OOH^−^) with consequent hydroxylation of L-arginine, resulting in the formation of NOHA and regeneration of Fe^3+^ from heme group. In the next step, NOHA participates in another oxidation-reduction cycle by binding to Fe^3+^, which will receive more electrons from the reductase group, resulting in the cleavage of NOHA and release of water, L-citrulline and NO (Abu-Soud et al., 1994, 2000; Rosen et al., 2002).

Therefore, during the enzymatic formation of NO cycle, nNOS also generates H_2_O_2_ and ${\text{O}}_{2}^{•-}$ (Figure 1). The production of these reactive oxygen species (ROS) by nNOS can occur even at saturating concentrations of L-arginine or NOHA in steps before the formation of NO (Rosen et al., 2002; Tsai et al., 2005; Weaver et al., 2005). At the expense of ${\text{O}}_{2}^{•-}$, the production of H_2_O_2_ is strongly increased by BH_4_ (Rosen et al., 2002).

## Role of nNOS in vascular homeostasis

Emerging evidence shows that nNOS has a physiologically relevant role in the control of the cardiovascular system. Here, we outline the recent advances on the role of nNOS in the vascular function.

There are several reports implicating the participation of nNOS in cerebral blood flow (CBF; Pelligrino et al., 1993; Santizo et al., 2000; Chi et al., 2003). Intraperitoneal injections of the selective nNOS inhibitor 7-nitroindazole (7-NI) depressed baseline CBF in rats (Montécot et al., 1997; Gotoh et al., 2001). Moreover, 7-NI decreased cerebral capillary flow in rats (Hudetz et al., 1998) and global CBF in cats (Hayashi et al., 2002). In rats, during hyperbaric conditions, it was found that the increase in CBF in the cortex prior to the appearance of electrical discharges was completely inhibited by 7-NI (Hagioka et al., 2005).

Aside from cerebral flow, it has been suggested that nNOS-derived NO regulates renal circulation. In the presence of, S-methyl-L-thiocitrulline (SMTC) a nNOS inhibitor, the vasoconstrictor response to angiotensin II is increased in the efferent arteriole (Ichihara et al., 1998). Additional evidence was obtained from nNOS^−∕−^ mice, where genetic deletion of nNOS decreases medullary blood flow in response to angiotensin II (Mattson and Meister, 2005). In nNOS^−∕−^ mice Vallon et al. (2001) also found that the feedback control of glomerular vascular tone is attenuated.

Similarly, studies in isolated vessels demonstrate the participation of nNOS in the control of vascular function. In pial arterioles of eNOS^−∕−^ mice acetylcholine induced an nNOS-cGMP-dependent vasodilation (Meng et al., 1996, 1998). Another work confirmed the presence of nNOS in the endothelium of coronary arteries of eNOS^−∕−^ mice and showed that shear stress activated endothelial nNOS-derived NO release, compensating the absence of eNOS-derived NO (Huang et al., 2002). In aorta of nNOS^−∕−^ mice the vasodilator response induced by acetylcholine is reduced (Nangle et al., 2004). In small mesenteric arteries of female rats the inhibition of endothelial nNOS contributes to the decrease in the relaxation induced by estrogen. Furthermore, the same study showed that estrogen rapidly increased the nNOS activity and nNOS-mediated NO production in human umbilical vein endothelial cells (Lekontseva et al., 2011). A year later, the same group demonstrated that nNOS contributed to the estrogen-mediated vascular relaxation of mesenteric artery in young, but not in ovariectomized and aging female rats. In the ovariectomized and aging group nNOS functionally became a source of ${\text{O}}_{2}^{•-}$ (Lekontseva et al., 2012).

Corroborating the above findings, NO release from nNOS also seems to be important in the control of vascular tone in humans. Expression of nNOS was found in human aorta, carotid, radial and mammary artery (Buchwalow et al., 2002), saphenous vein (Webb et al., 2006), and lung capillary endothelial cells (Lührs et al., 2002).

The first evidence that nNOS had a function in vascular regulation in humans was obtained from children suffering from Duchene muscular dystrophy (DMD). It was shown that nNOS-derived NO present in skeletal muscle acts in the blood flow and oxygen transport. nNOS expression is reduced in children with DMD resulting in increased vasoconstrictor response (Sander et al., 2000).

Later on, Seddon et al. (2008) showed the relationship between nNOS and the regulation of blood flow in human. Selective *in vivo* inhibition of nNOS with SMTC in healthy men promoted a reduction in the brachial artery baseline flow. This effect was eliminated in the presence of L-arginine. A similar reduction was observed with the non-selective inhibitor of NOS (L-NMMA) but required a 20-fold higher dose. This study suggested that nNOS-derived NO has a significant role in the physiological regulation of microvascular tone *in vivo* (Seddon et al., 2008). In another work, the same group investigated the *in vivo* effects of SMTC in human coronary dilatation. The infusion of SMTC in healthy patients reduced baseline coronary blood flow and coronary artery diameter measured by angiography. They concluded that local nNOS-derived NO is a key physiological regulator of human coronary vascular tone *in vivo* (Seddon et al., 2009).

All the above works suggesting NO as the mediator of nNOS function in the regulation of vascular tone were based on the assumption that NO was the only physiological vasodilator product of nNOS activation. Our group was the first to show the importance of nNOS-derived H_2_O_2_ in the endothelium-dependent vascular relaxation. We showed that nNOS was constitutively expressed in the endothelium of the mouse aorta and mesenteric resistance artery. Stimulation of those vessels with acetylcholine promoted increase in H_2_O_2_ production. Pharmacological selective nNOS inhibition and nNOS knockdown decreased endothelium-dependent vascular relaxation and H_2_O_2_ production. Finally, incubation of the vessels with catalase, an enzyme that degrades H_2_O_2_ into O_2_ and H_2_O, decreased vascular relaxation (Capettini et al., 2008, 2010; Silva et al., 2016). The participation of nNOS in vascular homeostasis in physiological and pathological conditions is summarized in Table 1.

**Table 1:** **Participation of nNOS in the control of vascular function in physiological conditions and during hypertension and atherosclerosis**.

**Vascular tissue**	**Effect**	**Model**	**References**
**PHYSIOLOGICAL CONDITIONS**			
Internal thoracic artery, saphenous vein, aorta, carotid artery, pancreas arterioles, and venous	nNOS expression in vascular smooth muscle and endothelial cells	Human	Buchwalow et al., 2002; Webb et al., 2006
Mammary artery	nNOS expression in smooth muscle cells	Human	Buchwalow et al., 2002
Pulmonary capillary	nNOS expression in endothelial cells	Human	Lührs et al., 2002
Brachial and coronary artery	nNOS inhibition decreases baseline flow	Human	Seddon et al., 2008, 2009
Aorta	Reduction of Acetylcholine-induced vasodilation	nNOS^−∕−^mice	Nangle et al., 2004
	nNOS-derived H_2_O_2_ contributes to endothelium-dependent vascular relaxation	Mice	Capettini et al., 2008, 2010
Pial arteriole	Acetylcholine-induced nNOS-cGMP-dependent vasodilation	eNOS^−∕−^ mice	Meng et al., 1996, 1998
Renal cortical and medullary blood vessels	Decrease in medullary blood flow in response to angiotensin II	nNOS^−∕−^mice	Mattson and Meister, 2005
Coronary artery	Endothelial nNOS-derived NO maintains flow-induced dilation	eNOS^−∕−^ mice	Huang et al., 2002
Glomerular vessels	Attenuation of the feedback control of glomerular vascular tone	nNOS^−∕−^mice	Vallon et al., 2001
Mesenteric artery	nNOS participates in estrogen-induced relaxation	Female Rats	Lekontseva et al., 2011, 2012
Renal efferent arteriole	nNOS inhibition increases the vasoconstrictor response to angiotensin II	Rats	Ichihara et al., 1998
Cerebral vasculature	nNOS inhibition decreases cerebral blood flow	Rats	Santizo et al., 2000; Gotoh et al., 2001; Chi et al., 2003; Hagioka et al., 2005
Cerebral vasculature	nNOS inhibition decreases cerebral blood flow	Cats	Hayashi et al., 2002
**HYPERTENSION**			
Aorta and *in vivo* experiments	nNOS inhibition decreases vascular tone and increases blood pressure in normotensive but not in SHR	Rats	Cacanyiova et al., 2009, 2012
Carotid artery	Increase in nNOS expression and functioning	SHR	Boulanger et al., 1998
Mesenteric artery	Increase in nNOS expression	SHR	Briones et al., 2000
	Decrease in nNOS-derived NO bioavailability in old animals	SHR	Ferrer et al., 2003
	impairment of nNOS-derived H_2_O_2_ production contributes to endothelial dysfunction	DOCA-salt-hypertensive mice	Silva et al., 2016
**ATHEROSCLEROSIS**			
Aorta	Increase in atherosclerotic plaque formation	apoE^−∕−^ nNOS^−∕−^ double knockout mice	Kuhlencordt et al., 2006
	nNOS-derived H_2_O_2_ contributes to endothelial dysfunction	apoE^−∕−^ mice	Capettini et al., 2011
	nNOS mRNA is expressed in atherosclerotic lesions	Human	Wilcox et al., 1997
Carotid artery	nNOS accelerates neointimal formation and constrictive vascular remodeling	Carotid artery ligation in nNOS^−∕−^ mice and rat balloon injury model	Morishita et al., 2002
	nNOS gene therapy decreases markers of atherosclerosis	Cholesterol-fed rabbit	Qian et al., 1999

*SHR, spontaneously hypertensive rats*.

## nNOS in vascular diseases

### Hypertension

Several studies have indicated that the imbalance in nNOS expression and/or activity is involved in the mechanism of pathogenesis of hypertension. In mesenteric arteries from spontaneously hypertensive rats (SHR), nNOS expression was ~2 times higher than in vessels from control animals (Briones et al., 2000). A similar result showing increased expression of nNOS in vascular smooth muscle cells was found in carotid arteries from SHR. It was shown that activation of nNOS on stimulation by Angiotensin II occurs in hypertensive but not in normotensive animals (Boulanger et al., 1998). Interestingly, in SHR rats the expression and activity of nNOS are decreased in the adrenal gland. Chronic treatment of SHR with antihypertensive drugs, increased the expression and activity of nNOS in the adrenal gland, suggesting that normalization of blood pressure (BP) may be in part related to an increase in nNOS (Qadri et al., 2001).

BP and vascular function were evaluated in normotensive rats chronically treated (6 weeks) with the selective nNOS inhibitor 7-NI. A significant increase in systolic BP was observed in the first 2 weeks of treatment. Corroborating the *in vivo* study, isolated vessels showed an attenuated relaxant response to acetylcholine in the aorta. These results show that nNOS participates in the regulation of BP and vascular tone (Cacanyiova et al., 2009). In contrast, in SHR, treatment with 7-NI had no effect in blood pressure or acetylcholine-induced vasodilatation in the aorta (Cacanyiova et al., 2009, 2012), suggesting that nNOS function was lost in hypertension.

A recent study revealed that impairment of nNOS-derived H_2_O_2_ pathway participates in the endothelial dysfunction and increase in blood pressure in DOCA-salt-hypertensive mice (Silva et al., 2016). This study showed that 1-(2-trifluoromethylphenyl) imidazole, a selective nNOS inhibitor, and catalase, exhibited a more pronounced reduction of acetylcholine-induced decrease in blood pressure in normotensive than in hypertensive mice. Moreover, selective nNOS inhibition and catalase had a greater inhibitory effect in acetylcholine-induced vasodilatation in control compared to DOCA-salt mice. Also, acetylcholine-induced H_2_O_2_ production and the expression and functioning of nNOS were considerably diminished in the resistance mesenteric arteries of DOCA-salt mice.

### Atherosclerosis

The first evidence that nNOS plays a vasculoprotective role in atherosclerosis came from a work by Wilcox et al. (1997) that showed a correlation between the progression of plaque formation and nNOS mRNA. In 1999, Qian et al. performed experiments with recombinant adenoviruses expressing nNOS transferred to carotid of hypercholesterolemic rabbits and showed a marked reduction in expression of adhesion molecules and infiltration of inflammatory cells. Additionally, a reduction in lipid deposition was observed after gene transfer. In another work, nNOS^−∕−^ mice exhibited accelerated neointimal formation and constrictive vascular remodeling caused by blood flow disruption in a model of carotid artery ligation. It was also observed that selective inhibition of nNOS decreased cGMP production, inducing an increase in vasoconstrictor response and accelerating neointimal formation in a rat balloon injury model (Morishita et al., 2002). Using a double knockout mouse (nNOS DKO) that combined genetic deletion of nNOS (nNOS^−∕−^) with a model of atherosclerosis (apoE^−∕−^), Kuhlencordt et al. (2006) showed that the absence of nNOS accelerated the atherosclerotic plaque lesion. After 14 weeks following a “Western-type” atherogenic diet, nNOS DKO animals showed 66% increase of lesion area, compared to apoE^−∕−^ control mice.

nNOS-derived H_2_O_2_ also seems to participate in endothelial dysfunction in atherosclerosis. Capettini et al. (2011) showed that selective pharmacological inhibition of nNOS, nNOS knockdown and catalase reduced the vasodilator effect of acetylcholine, diminished NO and abolished endothelial-dependent H_2_O_2_ production in wild-type mice, but had no effect in ApoE^−∕−^ animals. In addition, nNOS functioning was decreased in ApoE^−∕−^ mice compared to controls.

## Conclusions

This mini-review summarizes puzzling information on the role of nNOS in the control of vascular homeostasis under physiological and diseases conditions. Recent data indicates that nNOS is constitutively expressed in the endothelial cells of different types of vessels in animals and human. More importantly, nNOS-derived products such as NO and H_2_O_2_ play an important role in the control of vascular function and blood pressure. Finally, nNOS participates in the physiopathology of hypertension and atherosclerosis.

## Author contributions

VL defined the research topics and co-wrote the manuscript. EC, BR, and SC co-wrote the manuscript.

## Funding

This work was supported by FAPEMIG (Fundação de Apoio à Pesquisa do Estado de Minas Gerais) grant APQ-00683-13 and CNPq (Conselho Nacional de Desenvolvimento Científico e Tecnológico) grants 467147/2014-0, 305693/2014-0, and 470860/2012-0.

### Conflict of interest statement

The authors declare that the research was conducted in the absence of any commercial or financial relationships that could be construed as a potential conflict of interest.
